# A Sensitive Real-Time PCR-Based Assay for the Identification of *Colletotrichum* in Phytosanitary and Clinical Applications

**DOI:** 10.3390/jof12030171

**Published:** 2026-02-27

**Authors:** Dorji Phurbu, Weijun Duan, Pedro W. Crous, Lei Cai, Fang Liu

**Affiliations:** 1 Xizang Key Laboratory of Plateau Fungi, Institute of Plateau Biology of Xizang Autonomous Region, Lhasa 850001, China; 2 Ningbo Academy of Inspection and Quarantine, Ningbo 315012, China; 3 Technical Center of Ningbo Customs District, Ningbo 315012, China; 4 Westerdijk Fungal Biodiversity Institute, Uppsalalaan 8, 3584 CT Utrecht, The Netherlands; 5 Microbiology, Department of Biology, Utrecht University, Padualaan 8, 3584 CT Utrecht, The Netherlands; 6 Department of Biochemistry, Genetics and Microbiology, Forestry and Agricultural Biotechnology Institute (FABI), Faculty of Natural and Agricultural Sciences, University of Pretoria, Private Bag X20, Hatfield, Pretoria 0028, South Africa; 7 Laboratory of Phytopathology, Wageningen University and Research Centre (WUR), Droevendaalsesteeg 1, 6708 PB Wageningen, The Netherlands; 8 State Key Laboratory of Microbial Diversity and Innovative Utilization, Institute of Microbiology, Chinese Academy of Sciences, Beijing 100101, China; 9 College of Life Sciences, University of Chinese Academy of Sciences, Beijing 100049, China

**Keywords:** human pathogen, molecular diagnosis, plant pathogen, qPCR, specific primer

## Abstract

*Colletotrichum* species are major plant pathogens and emerging opportunistic human pathogens. Due to their vast genetic diversity, existing diagnostic tools often suffer from narrow specificity or labor-intensive workflows. In this study, we developed a rapid, universal, and highly sensitive genus-specific real-time PCR assay utilizing a TaqMan MGB probe targeting the conserved 28S rDNA region. The assay demonstrated exceptional specificity, with no cross-reactivity against closely related fungal taxa or common co-occurring pathogens. The method exhibited high sensitivity, achieving a limit of detection (LOD) of 680 fg of genomic DNA. Furthermore, the assay was successfully validated using simulated environmental samples, where it accurately identified *Colletotrichum* within complex fungal communities. By providing a robust platform for genus-level screening, this methodology significantly enhances the efficiency of phytosanitary inspections and clinical diagnostics, facilitating timely biosecurity interventions and therapeutic decisions.

## 1. Introduction

*Colletotrichum*, the sole genus in the family Glomerellaceae (Glomerellales), is widely recognized as one of the top 10 most important groups of plant pathogenic fungi globally [1]. Exhibiting a cosmopolitan distribution and an extensive host range, species within this genus infect a vast array of agricultural crops, economic trees, and ornamental plants, causing devastating anthracnose diseases. For instance, *C. kahawae*, the causal agent of Coffee Berry Disease, can result in yield losses of 30–70% or even total crop failure in African Arabica coffee production [2,3]. Consequently, it is listed as a priority quarantine pest in China, the European Union, and various other regions. Similarly, the average anthracnose disease incidence in chili peppers caused by *C. scovillei* (syn. *C. capsici*) was reported at 63%, with an average disease intensity of 68% in Indonesia [4]. High-value crops such as strawberries, mangoes, citrus, and avocados are particularly susceptible, facing severe quality degradation and economic downturns due to fruit rot [5]. Additionally, ubiquitous species complexes such as *C. gloeosporioides*, *C. acutatum*, and *C. boninense* are responsible for significant losses in forestry and agriculture worldwide [6,7,8]. Therefore, accurate identification and early warning of this genus are of profound economic and ecological importance.

Beyond their impact on agriculture, *Colletotrichum* species are emerging as opportunistic human pathogens. They are increasingly implicated in localized infections such as keratitis and phaeohyphomycosis (subcutaneous infections), as well as systemic diseases, particularly in immunocompromised individuals or following traumatic inoculation [9,10]. Clinically relevant species include *C. chlorophyti* [11], *C. coccodes* [12], *C. dematium* [13,14], *C. gigasporum* [15], *C. gloeosporioides* [14], *C. siamense* [16], and *C. truncatum* [17]. These infections are often linked to direct contact with infected plant material or contaminated soil, posing a growing “One Health” public health risk that necessitates vigilance [18].

As one of the fungal groups most frequently intercepted at ports of entry, *Colletotrichum* poses a significant challenge to customs and phytosanitary authorities. Rapid screening and interception serve as the first line of defense against biological invasions. However, the taxonomy of *Colletotrichum* has undergone significant revision, with the genus now organized into approximately 17 species complexes and several singleton species [5,6,7,19]. Currently, routine identification in quarantine and clinical laboratories relies on a traditional workflow involving isolation, morphological observation, and multi-locus (e.g., ITS, *cal*, *gapdh*, *gs*, *his3*, *tub2*, and *ApMat*) phylogenetic analysis [19]. While robust, this approach is labor-intensive, time-consuming, and requires specialized taxonomic expertise, failing to meet the demand for high-throughput, rapid-turnaround testing required for efficient customs clearance and clinical diagnosis. Similarly, while High-Throughput Sequencing (NGS) offers powerful capabilities for analyzing environmental samples, its high cost, long cycle and requirement for complex bioinformatic analysis hinder its routine deployment in frontline inspection.

In terms of molecular diagnostics in *Colletotrichum*, various methods, including conventional PCR, real-time PCR and Loop-Mediated Isothermal Amplification (LAMP), have been developed. However, the majority of these assays are designed for specific species (e.g., *C. acutatum* or *C. kahawae*) [20,21]. Given the high diversity and genetic complexity within the genus, single-species assays are inefficient for initial screening and are prone to false negatives when dealing with unknown or mixed infections. Consequently, existing tools cannot satisfy the practical need for a “genus-level” screen. Therefore, there is an urgent need to develop a rapid, sensitive, and universal molecular detection method capable of covering the vast majority of *Colletotrichum* species, thereby enhancing the efficiency of quarantine inspection and clinical pathogen screening.

Attempts to develop genus-specific primers have been made, but limitations persist. Mosca et al. [22] designed the degenerate genus-specific primers Coll1F/Coll3Rb based on ITS sequences; however, their specificity validation relied on a limited dataset. By aligning their primer sequences against our comprehensive *Colletotrichum* ITS dataset [19], we identified a single-nucleotide mismatch in the reverse primer in species belonging to the *C. orbiculare* species complex (e.g., *C. lindemuthianum*, *C. orbiculare*, *C. trifolii*), suggesting potential detection failures for these taxa. Similarly, Li et al. [23] developed the ITS-based primers ITS-c3/ITS-c4. Sequence alignment against our dataset revealed a single-nucleotide mismatch in the forward primer against *C. ocimi* and *C. pseudoacutatum*, as well as multiple mismatches in the reverse primer across several species, indicating that the detection efficiency of this set may be compromised. Furthermore, these methods rely on conventional PCR followed by gel electrophoresis, which is less efficient than real-time quantitative PCR (qPCR) platforms.

Consequently, there is an urgent need to develop a rapid and reliable molecular technique for the universal detection of the genus *Colletotrichum*. The objective of this study was to establish and validate a novel, genus-specific TaqMan real-time PCR assay. The specificity and robustness of this assay were rigorously evaluated using a comprehensive panel of isolates, which covered the majority of *Colletotrichum* species complexes (including those reported as human pathogens and serious phytopathogens), closely related fungal families, and other common phytopathogens. Furthermore, the assay’s effectiveness was verified using simulated environmental samples containing complex fungal communities.

## 2. Materials and Methods

### 2.1. Fungal Strains and DNA Extraction

A total of 52 *Colletotrichum* and 28 non-*Colletotrichum* strains belonging to the orders Botryosphaeriales, Chaetosphaeriales, Diaporthales, Eurotiales, Hypocreales, Mycosphaerellales, and Pleosporales were used in this study (Table 1). Their DNA was extracted using a modified CTAB protocol, and DNA concentration was quantified using NanoDrop^TM^ One (Thermo Fisher Scientific, Waltham, MA, USA) [24]. The identities of all *Colletotrichum* isolates used were confirmed via multi-gene phylogenetic analysis as described in previous studies [8,15,19]. For non-*Colletotrichum* species, isolates labeled with “LC” (personal culture collection of Prof. Lei Cai, housed in the Institute of Microbiology, Chinese Academy of Sciences) were identified based on ITS sequence similarity, while DNA for isolates labeled with “CBS” was obtained from the Westerdijk Fungal Biodiversity Institute (Utrecht, The Netherlands). Furthermore, to validate the intra-generic conservation of the designed primers, an in silico validation was performed using 97 whole-genome sequences of *Colletotrichum* available in the NCBI database (Table 1).

### 2.2. 28S rDNA Sequencing and Design of Primers and Probe

Due to the limited availability of 28S rDNA sequences for *Colletotrichum* species in GenBank, we sequenced the 28S region of 29 *Colletotrichum* isolates maintained in our laboratory. PCR amplification was performed using the primers LR0R (5‘-ACCCGCTGAACTTAAGC-3′) and LR5 (5‘-ATCCTGAGGGAAACTTC-3′), with reaction systems and conditions following Liu et al. [19]. The obtained sequences were aligned with those of closely related species and common plant pathogens using MEGA 7.0 software.

**Figure 1:**
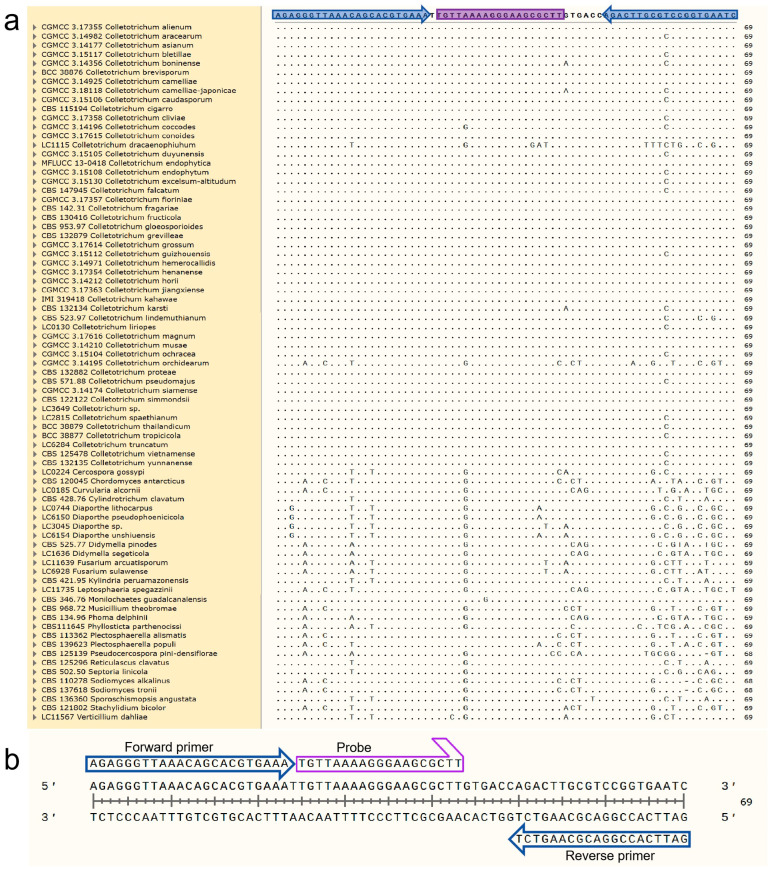
Alignment of 28S sequences of *Colletotrichum* spp. and other genera. (**a**) Location of forward and reverse primers (blue arrows) and qPCR probe (in purple) designed for the detection of *Colletotrichum* spp. (**b**) Detailed schematic of how target gene fragments bind.

Nucleotide sequence regions exhibiting a consensus specific to *Colletotrichum* spp. were selected for primer and probe design using Primer Express 3.0 software (Applied Biosystems, Foster City, CA, USA). The resulting assay utilizes the forward primer CLF (5′-AGAGGGTTAAACAGCACGTGAAA-3′), the reverse primer CLR (5′-GATTCACCGGACGCAAGTCT-3′), and the TaqMan probe CLP (5′-FAM-TGTTAAAAGGGAAGCGCTT-MGB-3′), generating a 69 bp amplicon (Figure 1). The Primer Express 3.0.1 software was employed to check for hairpins and potential seco. Specificity was further verified in silico using the BLASTn tool of the National Center for Biotechnology Information (https://blast.ncbi.nlm.nih.gov/Blast.cgi, (accessed on 20 December 2025)). The primers and the probe were synthesized by Invitrogen. The TaqMan^®^ probe was labeled with 6-FAM (fluorescein) at the 5′ end and a non-fluorescent quencher (NFQ) coupled with a Minor Groove Binder (MGB) moiety at the 3′ end.

**Table 1:** Strains or data used in this study.

Genus	Epithet	Complex ^1^	Family	Order	Strain No. ^2^	28S GenBank Accession No.	Genome Accession No.	Usage ^3^	qPCR Test ^4^
*Colletotrichum*	*abscissum*	Acutatum	Glomerellaceae	Glomerellales	IMI 504890	MOOD01000057.1	GCA_030869225.1	NCBI blast, 100% similarity	/
*Colletotrichum*	*acutatum*	Acutatum	Glomerellaceae	Glomerellales	Ca28	JBMPGZ010000235.1	GCA_000319635.1	NCBI blast, 100% similarity	/
*Colletotrichum*	*acutatum*	Acutatum	Glomerellaceae	Glomerellales	Ca34	JBMPGY010000508.1	GCA_049999025.1	NCBI blast, 100% similarity	/
*Colletotrichum*	*acutatum*	Acutatum	Glomerellaceae	Glomerellales	Ca40	JBMPGX010000441.1	GCA_049998925.1	NCBI blast, 100% similarity	/
*Colletotrichum*	*acutatum*	Acutatum	Glomerellaceae	Glomerellales	Ca6	JBMPHA010000508.1	GCA_049999125.1	NCBI blast, 100% similarity	/
*Colletotrichum*	*acutatum*	Acutatum	Glomerellaceae	Glomerellales	COL14	JAZDWV010000028.1	GCA_040760105.1	NCBI blast, 100% similarity	/
*Colletotrichum*	*acutatum*	Acutatum	Glomerellaceae	Glomerellales	COL14	JAZDWV010000018.1	GCA_040760105.1	NCBI blast, 100% similarity	/
*Colletotrichum*	*acutatum*	Acutatum	Glomerellaceae	Glomerellales	LC3649	/	/	Primer design & qPCR test	+
*Colletotrichum*	*aenigma*	Gloeosporioides	Glomerellaceae	Glomerellales	Cg56	QPNB01000015.1	GCA_013390185.1	NCBI blast, 100% similarity	/
*Colletotrichum*	*aenigma*	Gloeosporioides	Glomerellaceae	Glomerellales	Cg56	QPNB01000040.1	GCA_013390185.1	NCBI blast, 100% similarity	/
*Colletotrichum*	*aenigma*	Gloeosporioides	Glomerellaceae	Glomerellales	Cg56	QPNB01000043.1	GCA_013390185.1	NCBI blast, 100% similarity	/
*Colletotrichum*	*aenigma*	Gloeosporioides	Glomerellaceae	Glomerellales	Cg56	QPNB01000058.1	GCA_013390185.1	NCBI blast, 100% similarity	/
*Colletotrichum*	*aenigma*	Gloeosporioides	Glomerellaceae	Glomerellales	Cg56	QPNB01000065.1	GCA_013390185.1	NCBI blast, 100% similarity	/
*Colletotrichum*	*aenigma*	Gloeosporioides	Glomerellaceae	Glomerellales	Cg56	QPNB01000004.1	GCA_013390185.1	NCBI blast, 100% similarity	/
*Colletotrichum*	*aenigma*	Gloeosporioides	Glomerellaceae	Glomerellales	Cg56	QPNB01000019.1	GCA_013390185.1	NCBI blast, 100% similarity	/
*Colletotrichum*	*aenigma*	Gloeosporioides	Glomerellaceae	Glomerellales	Cg56	QPNB01000052.1	GCA_013390185.1	NCBI blast, 100% similarity	/
*Colletotrichum*	*aenigma*	Gloeosporioides	Glomerellaceae	Glomerellales	Cg56	QPNB01000057.1	GCA_013390185.1	NCBI blast, 100% similarity	/
*Colletotrichum*	*alienum*	Gloeosporioides	Glomerellaceae	Glomerellales	CGMCC 3.17355	/	/	Primer design & qPCR test	+
*Colletotrichum*	*aracearum*	Orchidearum	Glomerellaceae	Glomerellales	CGMCC 3.14982	/	/	Primer design & qPCR test	+
*Colletotrichum*	*asianum*	Gloeosporioides	Glomerellaceae	Glomerellales	CGMCC 3.14177	/	/	Primer design & qPCR test	+
*Colletotrichum*	*bletillae*	Spaethianum	Glomerellaceae	Glomerellales	CGMCC 3.15117	/	/	Primer design & qPCR test	+
*Colletotrichum*	*boninense*	Boninense	Glomerellaceae	Glomerellales	CGMCC 3.14356	/	/	Primer design & qPCR test	+
*Colletotrichum*	*brevisporum*	Magnum	Glomerellaceae	Glomerellales	BCC 38876	/	/	Primer design & qPCR test	+
*Colletotrichum*	*camelliae*	Gloeosporioides	Glomerellaceae	Glomerellales	CGMCC 3.14925	/	/	Primer design & qPCR test	+
*Colletotrichum*	*camelliae-japonicae*	Boninense	Glomerellaceae	Glomerellales	CGMCC 3.18118	/	/	Primer design & qPCR test	+
*Colletotrichum*	*caudasporum*	Caudatum	Glomerellaceae	Glomerellales	CGMCC 3.15106	/	/	Primer design & qPCR test	+
*Colletotrichum*	*cigarro*	Gloeosporioides	Glomerellaceae	Glomerellales	CBS 115194	/	/	Primer design & qPCR test	+
*Colletotrichum*	*cliviicola*	Orchidearum	Glomerellaceae	Glomerellales	CGMCC 3.17358	/	/	Primer design & qPCR test	+
*Colletotrichum*	*coccodes*	/	Glomerellaceae	Glomerellales	CGMCC 3.14196	/	/	Primer design	/
*Colletotrichum*	*conoides*	Gloeosporioides	Glomerellaceae	Glomerellales	CGMCC 3.17615	/	/	Primer design & qPCR test	+
*Colletotrichum*	*costaricense*	Acutatum	Glomerellaceae	Glomerellales	IMI 309622	MOOE01000009.1	GCA_030867565.1	NCBI blast, 100% similarity	/
*Colletotrichum*	*cuscutae*	Acutatum	Glomerellaceae	Glomerellales	IMI 304802	MPDP01000298.1	GCA_030869315.1	NCBI blast, 100% similarity	/
*Colletotrichum*	*dematium*	Dematium	Glomerellaceae	Glomerellales	LC3659	/	/	Primer design & qPCR test	+
*Colletotrichum*	*destructivum*	Destructivum	Glomerellaceae	Glomerellales	YC1	WWFS01000028.1	GCA_009900065.1	NCBI blast, 100% similarity	/
*Colletotrichum*	*destructivum*	Destructivum	Glomerellaceae	Glomerellales	YC1	WWFS01000044.1	GCA_009900065.1	NCBI blast, 100% similarity	/
*Colletotrichum*	*dracaenophilum*	Dracaenophilum	Glomerellaceae	Glomerellales	LC1115	/	/	Primer design & qPCR test	+
*Colletotrichum*	*duyunensis*	Caudatum	Glomerellaceae	Glomerellales	CGMCC 3.15105	/	/	Primer design & qPCR test	+
*Colletotrichum*	*endophyticum*	Gloeosporioides	Glomerellaceae	Glomerellales	MFLUCC 13-0418	/	/	Primer design & qPCR test	+
*Colletotrichum*	*endophytum*	Graminicola	Glomerellaceae	Glomerellales	CGMCC 3.15108	/	/	Primer design & qPCR test	+
*Colletotrichum*	*excelsum-altitudum*	Dracaenophilum	Glomerellaceae	Glomerellales	CGMCC 3.15130	/	/	Primer design & qPCR test	+
*Colletotrichum*	*falcatum*	Graminicola	Glomerellaceae	Glomerellales	CBS 147945	/	/	Primer design & qPCR test	+
*Colletotrichum*	*filicis*	Acutatum	Glomerellaceae	Glomerellales	CBS 101611	MOOC01000267.1	GCA_023376865.1	NCBI blast, 100% similarity	/
*Colletotrichum*	*fioriniae*	Acutatum	Glomerellaceae	Glomerellales	CGMCC 3.17357	/	/	Primer design & qPCR test	+
*Colletotrichum*	*fragariae*	Gloeosporioides	Glomerellaceae	Glomerellales	CBS 142.31	/	/	Primer design & qPCR test	+
*Colletotrichum*	*fructicola*	Gloeosporioides	Glomerellaceae	Glomerellales	1104-7	MVNS02000003.1	GCA_002314275.2	NCBI blast, 100% similarity	/
*Colletotrichum*	*fructicola*	Gloeosporioides	Glomerellaceae	Glomerellales	CBS 130416	/	/	Primer design & qPCR test	+
*Colletotrichum*	*fructicola*	Gloeosporioides	Glomerellaceae	Glomerellales	Cf245	QPMY01000297.1	GCA_013201925.1	NCBI blast, 100% similarity	/
*Colletotrichum*	*fructicola*	Gloeosporioides	Glomerellaceae	Glomerellales	Cf413	QPMX01000004.1	GCA_013390205.1	NCBI blast, 100% similarity	/
*Colletotrichum*	*fructicola*	Gloeosporioides	Glomerellaceae	Glomerellales	Cf413	QPMX01000013.1	GCA_013390205.1	NCBI blast, 100% similarity	/
*Colletotrichum*	*fructicola*	Gloeosporioides	Glomerellaceae	Glomerellales	Cf415	QPMW01000284.1	GCA_013201905.1	NCBI blast, 100% similarity	/
*Colletotrichum*	*fructicola*	Gloeosporioides	Glomerellaceae	Glomerellales	Cg38 S1	QLYQ01000011.1	GCA_012932255.1	NCBI blast, 100% similarity	/
*Colletotrichum*	*fructicola*	Gloeosporioides	Glomerellaceae	Glomerellales	CGMCC 3.17371	SSNE01000200.1	GCA_009771025.1	NCBI blast, 100% similarity	/
*Colletotrichum*	*fructicola*	Gloeosporioides	Glomerellaceae	Glomerellales	CZ2101	JBJJOW010000571.1	GCA_046120875.1	NCBI blast, 100% similarity	/
*Colletotrichum*	*fructicola*	Gloeosporioides	Glomerellaceae	Glomerellales	PZ02	JBMAIS010001167.1	GCA_048771815.1	NCBI blast, 100% similarity	/
*Colletotrichum*	*gloeosporioides*	Gloeosporioides	Glomerellaceae	Glomerellales	CBS 953.97	/	/	Primer design & qPCR test	+
*Colletotrichum*	*gloeosporioides*	Gloeosporioides	Glomerellaceae	Glomerellales	Cg28	JBBLNQ010000499.1	GCA_040955545.1	NCBI blast, 100% similarity	/
*Colletotrichum*	*gloeosporioides*	Gloeosporioides	Glomerellaceae	Glomerellales	Cg57	JBBLNP010000559.1	GCA_040955525.1	NCBI blast, 100% similarity	/
*Colletotrichum*	*gloeosporioides*	Gloeosporioides	Glomerellaceae	Glomerellales	Cg58	JBMPGW010000524.1	GCA_049998905.1	NCBI blast, 100% similarity	/
*Colletotrichum*	*gloeosporioides*	Gloeosporioides	Glomerellaceae	Glomerellales	Cg84	JBBLNO010000541.1	GCA_040955925.1	NCBI blast, 100% similarity	/
*Colletotrichum*	*gloeosporioides*	Gloeosporioides	Glomerellaceae	Glomerellales	COLG-95	WEZO01002064.1	GCA_011428055.1	NCBI blast, 100% similarity	/
*Colletotrichum*	*gloeosporioides*	Gloeosporioides	Glomerellaceae	Glomerellales	TYU	NOWE01000054.1	GCA_002901105.1	NCBI blast, 100% similarity	/
*Colletotrichum*	*gloeosporioides*	Gloeosporioides	Glomerellaceae	Glomerellales	TYU	NOWE01000056.1	GCA_002901105.1	NCBI blast, 100% similarity	/
*Colletotrichum*	*grevilleae*	Gloeosporioides	Glomerellaceae	Glomerellales	CBS 132879	/	/	Primer design & qPCR test	+
*Colletotrichum*	*grossum*	Gloeosporioides	Glomerellaceae	Glomerellales	CGMCC 3.17614	/	/	Primer design & qPCR test	+
*Colletotrichum*	*guizhouensis*	Spaethianum	Glomerellaceae	Glomerellales	CGMCC 3.15112	/	/	Primer design & qPCR test	+
*Colletotrichum*	*hemerocallidis*	Dematium	Glomerellaceae	Glomerellales	CGMCC 3.14971	/	/	Primer design & qPCR test	+
*Colletotrichum*	*henanense*	Gloeosporioides	Glomerellaceae	Glomerellales	CGMCC 3.17354	/	/	Primer design & qPCR test	+
*Colletotrichum*	*higginsianum*	Destructivum	Glomerellaceae	Glomerellales	IMI 349063	LTAN01000015.1	GCF_001672515.1	NCBI blast, 100% similarity	/
*Colletotrichum*	*higginsianum*	Destructivum	Glomerellaceae	Glomerellales	IMI 349063	LTAN01000019.1	GCF_001672515.1	NCBI blast, 100% similarity	/
*Colletotrichum*	*higginsianum*	Destructivum	Glomerellaceae	Glomerellales	IMI 349063	LTAN01000020.1	GCF_001672515.1	NCBI blast, 100% similarity	/
*Colletotrichum*	*higginsianum*	Destructivum	Glomerellaceae	Glomerellales	IMI 349063	LTAN01000022.1	GCF_001672515.1	NCBI blast, 100% similarity	/
*Colletotrichum*	*higginsianum*	Destructivum	Glomerellaceae	Glomerellales	IMI 349063	LTAN01000024.1	GCF_001672515.1	NCBI blast, 100% similarity	/
*Colletotrichum*	*higginsianum*	Destructivum	Glomerellaceae	Glomerellales	IMI 349063	LTAN01000007.1	GCF_001672515.1	NCBI blast, 100% similarity	/
*Colletotrichum*	*higginsianum*	Destructivum	Glomerellaceae	Glomerellales	IMI 349063	LTAN01000013.1	GCF_001672515.1	NCBI blast, 100% similarity	/
*Colletotrichum*	*higginsianum*	Destructivum	Glomerellaceae	Glomerellales	IMI 349063	LTAN01000014.1	GCF_001672515.1	NCBI blast, 100% similarity	/
*Colletotrichum*	*higginsianum*	Destructivum	Glomerellaceae	Glomerellales	IMI 349063	LTAN01000016.1	GCF_001672515.1	NCBI blast, 100% similarity	/
*Colletotrichum*	*higginsianum*	Destructivum	Glomerellaceae	Glomerellales	IMI 349063	LTAN01000017.1	GCF_001672515.1	NCBI blast, 100% similarity	/
*Colletotrichum*	*higginsianum*	Destructivum	Glomerellaceae	Glomerellales	IMI 349063	LTAN01000018.1	GCF_001672515.1	NCBI blast, 100% similarity	/
*Colletotrichum*	*higginsianum*	Destructivum	Glomerellaceae	Glomerellales	IMI 349063	LTAN01000021.1	GCF_001672515.1	NCBI blast, 100% similarity	/
*Colletotrichum*	*higginsianum*	Destructivum	Glomerellaceae	Glomerellales	IMI 349063	LTAN01000023.1	GCF_001672515.1	NCBI blast, 100% similarity	/
*Colletotrichum*	*higginsianum*	Destructivum	Glomerellaceae	Glomerellales	IMI 349063	LTAN01000025.1	GCF_001672515.1	NCBI blast, 100% similarity	/
*Colletotrichum*	*higginsianum*	Destructivum	Glomerellaceae	Glomerellales	MAFF 305635-RFP	MWPZ01000011.1	GCA_004920355.1	NCBI blast, 100% similarity	/
*Colletotrichum*	*higginsianum*	Destructivum	Glomerellaceae	Glomerellales	MAFF 305635-RFP	MWPZ01000019.1	GCA_004920355.1	NCBI blast, 100% similarity	/
*Colletotrichum*	*higginsianum*	Destructivum	Glomerellaceae	Glomerellales	MAFF 305635-RFP	MWPZ01000020.1	GCA_004920355.1	NCBI blast, 100% similarity	/
*Colletotrichum*	*horii*	Gloeosporioides	Glomerellaceae	Glomerellales	CGMCC 3.14212	/	/	Primer design & qPCR test	+
*Colletotrichum*	*hymenocallidis*	Gloeosporioides	Glomerellaceae	Glomerellales	CGMCC 3.14198, CBS 125378	/	/	Primer design & qPCR test	+
*Colletotrichum*	*jiangxiense*	Gloeosporioides	Glomerellaceae	Glomerellales	CGMCC 3.17363	/	/	Primer design & qPCR test	+
*Colletotrichum*	*kahawae*	Gloeosporioides	Glomerellaceae	Glomerellales	CIFC	VYYT01000170.1	GCA_032988895.1	NCBI blast, 100% similarity	/
*Colletotrichum*	*kahawae*	Gloeosporioides	Glomerellaceae	Glomerellales	IMI 319418	/	/	Primer design & qPCR test	+
*Colletotrichum*	*karsti*	Boninense	Glomerellaceae	Glomerellales	CGMCC 3.14194, CBS 132134	/	/	Primer design & qPCR test	+
*Colletotrichum*	*lindemuthianum*	Orbiculare	Glomerellaceae	Glomerellales	CBS 523.97	/	/	Primer design & qPCR test	+
*Colletotrichum*	*lini*	Destructivum	Glomerellaceae	Glomerellales	390-1	JAWQJW010000029.1	GCA_034638235.1	NCBI blast, 100% similarity	/
*Colletotrichum*	*lini*	Destructivum	Glomerellaceae	Glomerellales	390-1	JAWQJW010000030.1	GCA_034638235.1	NCBI blast, 100% similarity	/
*Colletotrichum*	*lini*	Destructivum	Glomerellaceae	Glomerellales	394-2	JBHKWU010000010.1	GCA_043790985.1	NCBI blast, 100% similarity	/
*Colletotrichum*	*liriopes*	Spaethianum	Glomerellaceae	Glomerellales	LC0130	/	/	Primer design & qPCR test	+
*Colletotrichum*	*magnum*	Magnum	Glomerellaceae	Glomerellales	CGMCC 3.17616	/	/	Primer design & qPCR test	+
*Colletotrichum*	*melonis*	Acutatum	Glomerellaceae	Glomerellales	Col 31	MLGG01000003.1	GCA_030868995.1	NCBI blast, 100% similarity	/
*Colletotrichum*	*musae*	Gloeosporioides	Glomerellaceae	Glomerellales	CGMCC 3.14210	/	/	Primer design & qPCR test	+
*Colletotrichum*	*musae*	Gloeosporioides	Glomerellaceae	Glomerellales	GM20	NWMS01009586.1	GCA_002814275.1	NCBI blast, 100% similarity	/
*Colletotrichum*	*ochracea*	Caudatum	Glomerellaceae	Glomerellales	CGMCC 3.15104	/	/	Primer design & qPCR test	+
*Colletotrichum*	*orchidearum*	Orchidearum	Glomerellaceae	Glomerellales	CGMCC 3.14195	/	/	Primer design & qPCR test	+
*Colletotrichum*	*orchidophilum*	singleton	Glomerellaceae	Glomerellales	IMI 309357	MJBS01000243.1	GCF_001831195.1	NCBI blast, 100% similarity	/
*Colletotrichum*	*proteae*	Gloeosporioides	Glomerellaceae	Glomerellales	CBS 132882	/	/	Primer design & qPCR test	+
*Colletotrichum*	*pseudomajus*	Gigasporum	Glomerellaceae	Glomerellales	CBS 571.88	/	/	Primer design & qPCR test	+
*Colletotrichum*	*scovillei*	Acutatum	Glomerellaceae	Glomerellales	KC05	LUXP02000007.1	GCA_001593745.2	NCBI blast, 100% similarity	/
*Colletotrichum*	*shisoi*	Destructivum	Glomerellaceae	Glomerellales	PG-2018a	PUHP01001126.1	GCA_006783085.1	NCBI blast, 100% similarity	/
*Colletotrichum*	*shisoi*	Destructivum	Glomerellaceae	Glomerellales	PG-2018a	PUHP01001576.1	GCA_006783085.1	NCBI blast, 100% similarity	/
*Colletotrichum*	*siamense*	Gloeosporioides	Glomerellaceae	Glomerellales	CAD1	QPMU01000156.1	GCA_013201865.1	NCBI blast, 100% similarity	/
*Colletotrichum*	*siamense*	Gloeosporioides	Glomerellaceae	Glomerellales	CAD2	QPMT01000145.1	GCA_013201745.1	NCBI blast, 100% similarity	/
*Colletotrichum*	*siamense*	Gloeosporioides	Glomerellaceae	Glomerellales	CAD4	QPMS01000144.1	GCA_013201795.1	NCBI blast, 100% similarity	/
*Colletotrichum*	*siamense*	Gloeosporioides	Glomerellaceae	Glomerellales	CAD5	QPMR01000158.1	GCA_013201755.1	NCBI blast, 100% similarity	/
*Colletotrichum*	*siamense*	Gloeosporioides	Glomerellaceae	Glomerellales	Cg363	QPNA01000004.1	GCF_013390195.1	NCBI blast, 100% similarity	/
*Colletotrichum*	*siamense*	Gloeosporioides	Glomerellaceae	Glomerellales	CGMCC 3.14174	/	/	Primer design & qPCR test	+
*Colletotrichum*	*siamense*	Gloeosporioides	Glomerellaceae	Glomerellales	CGMCC 3.14193, CBS130420	/	/	Primer design & qPCR test	+
*Colletotrichum*	*siamense*	Gloeosporioides	Glomerellaceae	Glomerellales	COLG-34	WEZJ01000519.1	GCA_011426385.1	NCBI blast, 100% similarity	/
*Colletotrichum*	*siamense*	Gloeosporioides	Glomerellaceae	Glomerellales	COLG-38	WEZK01000202.1	GCA_011426375.1	NCBI blast, 100% similarity	/
*Colletotrichum*	*siamense*	Gloeosporioides	Glomerellaceae	Glomerellales	COLG-44	WEZL01001991.1	GCA_011428115.1	NCBI blast, 100% similarity	/
*Colletotrichum*	*siamense*	Gloeosporioides	Glomerellaceae	Glomerellales	COLG-50	WEZM01001315.1	GCA_011428095.1	NCBI blast, 100% similarity	/
*Colletotrichum*	*siamense*	Gloeosporioides	Glomerellaceae	Glomerellales	COLG-90	WEZN01000556.1	GCA_011445115.1	NCBI blast, 100% similarity	/
*Colletotrichum*	*simmondsii*	Acutatum	Glomerellaceae	Glomerellales	CBS 122122	/	/	Primer design & qPCR test	+
*Colletotrichum*	*spaethianum*	Spaethianum	Glomerellaceae	Glomerellales	LC2815	/	/	Primer design & qPCR test	+
*Colletotrichum*	*tamarilloi*	Acutatum	Glomerellaceae	Glomerellales	Tom-12	MLFU01000212.1	GCA_030869305.1	NCBI blast, 100% similarity	/
*Colletotrichum*	*tanaceti*	Destructivum	Glomerellaceae	Glomerellales	BRIP 57314	PJEX01003345.1	GCA_005350895.1	NCBI blast, 100% similarity	/
*Colletotrichum*	*tanaceti*	Destructivum	Glomerellaceae	Glomerellales	BRIP 57314	PJEX01003605.1	GCA_005350895.1	NCBI blast, 100% similarity	/
*Colletotrichum*	*tanaceti*	Destructivum	Glomerellaceae	Glomerellales	BRIP 57314	PJEX01003839.1	GCA_005350895.1	NCBI blast, 100% similarity	/
*Colletotrichum*	*tanaceti*	Destructivum	Glomerellaceae	Glomerellales	BRIP 57314	PJEX01004016.1	GCA_005350895.1	NCBI blast, 100% similarity	/
*Colletotrichum*	*tanaceti*	Destructivum	Glomerellaceae	Glomerellales	BRIP 57314	PJEX01002211.1	GCA_005350895.1	NCBI blast, 100% similarity	/
*Colletotrichum*	*tanaceti*	Destructivum	Glomerellaceae	Glomerellales	BRIP 57314	PJEX01002212.1	GCA_005350895.1	NCBI blast, 100% similarity	/
*Colletotrichum*	*tanaceti*	Destructivum	Glomerellaceae	Glomerellales	BRIP 57314	PJEX01002213.1	GCA_005350895.1	NCBI blast, 100% similarity	/
*Colletotrichum*	*tanaceti*	Destructivum	Glomerellaceae	Glomerellales	BRIP 57314	PJEX01002215.1	GCA_005350895.1	NCBI blast, 100% similarity	/
*Colletotrichum*	*tanaceti*	Destructivum	Glomerellaceae	Glomerellales	BRIP 57314	PJEX01002219.1	GCA_005350895.1	NCBI blast, 100% similarity	/
*Colletotrichum*	*tanaceti*	Destructivum	Glomerellaceae	Glomerellales	BRIP 57314	PJEX01002221.1	GCA_005350895.1	NCBI blast, 100% similarity	/
*Colletotrichum*	*tanaceti*	Destructivum	Glomerellaceae	Glomerellales	BRIP 57314	PJEX01002222.1	GCA_005350895.1	NCBI blast, 100% similarity	/
*Colletotrichum*	*tanaceti*	Destructivum	Glomerellaceae	Glomerellales	BRIP 57314	PJEX01002367.1	GCA_005350895.1	NCBI blast, 100% similarity	/
*Colletotrichum*	*tanaceti*	Destructivum	Glomerellaceae	Glomerellales	BRIP 57314	PJEX01003061.1	GCA_005350895.1	NCBI blast, 100% similarity	/
*Colletotrichum*	*tanaceti*	Destructivum	Glomerellaceae	Glomerellales	BRIP 57314	PJEX01003647.1	GCA_005350895.1	NCBI blast, 100% similarity	/
*Colletotrichum*	*tanaceti*	Destructivum	Glomerellaceae	Glomerellales	BRIP 57314	PJEX01003690.1	GCA_005350895.1	NCBI blast, 100% similarity	/
*Colletotrichum*	*tanaceti*	Destructivum	Glomerellaceae	Glomerellales	BRIP 57314	PJEX01004933.1	GCA_005350895.1	NCBI blast, 100% similarity	/
*Colletotrichum*	*tanaceti*	Destructivum	Glomerellaceae	Glomerellales	BRIP 57314	PJEX01005191.1	GCA_005350895.1	NCBI blast, 100% similarity	/
*Colletotrichum*	*tanaceti*	Destructivum	Glomerellaceae	Glomerellales	BRIP 57315	RKSP01002127.1	GCA_026229895.1	NCBI blast, 100% similarity	/
*Colletotrichum*	*thailandicum*	Gloeosporioides	Glomerellaceae	Glomerellales	BCC 38879	/	/	Primer design & qPCR test	+
*Colletotrichum*	*tropicale*	Gloeosporioides	Glomerellaceae	Glomerellales	CgS9275	QPMP01000152.1	GCA_013201785.1	NCBI blast, 100% similarity	/
*Colletotrichum*	*tropicicola*	Dracaenophilum	Glomerellaceae	Glomerellales	BCC 38877	/	/	Primer design & qPCR test	+
*Colletotrichum*	*truncatum*	Truncatum	Glomerellaceae	Glomerellales	CMES1059	VUJX02000015.1	GCF_014235925.1	NCBI blast, 100% similarity	/
*Colletotrichum*	*truncatum*	Truncatum	Glomerellaceae	Glomerellales	KLC.C5	VRTN01000770.1	GCA_008131165.1	NCBI blast, 100% similarity	/
*Colletotrichum*	*truncatum*	Truncatum	Glomerellaceae	Glomerellales	LC6284	/	/	Primer design & qPCR test	+
*Colletotrichum*	*vietnamense*	Gigasporum	Glomerellaceae	Glomerellales	CBS 125478	/	/	Primer design & qPCR test	+
*Colletotrichum*	*viniferum*	Gloeosporioides	Glomerellaceae	Glomerellales	CGW01	QPMQ01000693.1	GCA_013201765.1	NCBI blast, 100% similarity	/
*Colletotrichum*	*yunnanense*	Dracaenophilum	Glomerellaceae	Glomerellales	CBS 132135	/	/	Primer design & qPCR test	+
*Cercospora*	*gossypii*	/	Mycosphaerellaceae	Mycosphaerellales	LC0224	/	/	Primer design & qPCR test	-
*Chordomyces*	*antarcticus*	/	Plectosphaerellaceae	Glomerellales	CBS 120045	/	/	Primer design & qPCR test	-
*Curvularia*	*alcornii*	/	Pleosporaceae	Pleosporales	LC0185	/	/	Primer design & qPCR test	-
*Cylindrotrichum*	*clavatum*	/	Reticulascaceae	Glomerellales	CBS 428.76	/	/	Primer design & qPCR test	-
*Diaporthe*	*lithocarpus*	/	Diaporthaceae	Diaporthales	LC0744	/	/	Primer design & qPCR test	-
*Diaporthe*	*pseudophoenicicola*	/	Diaporthaceae	Diaporthales	LC6150	/	/	Primer design & qPCR test	-
*Diaporthe*	sp.	/	Diaporthaceae	Diaporthales	LC3045	/	/	Primer design & qPCR test	-
*Diaporthe*	*unshiuensis*	/	Diaporthaceae	Diaporthales	LC6154	/	/	Primer design & qPCR test	-
*Didymella*	*pinodes*	/	Didymellaceae	Pleosporales	CBS 525.77	/	/	Primer design & qPCR test	-
*Fusarium*	*incarnatum*	/	Nectriaceae	Hypocreales	LC11639	/	/	Primer design & qPCR test	-
*Fusarium*	*sulawense*	/	Nectriaceae	Hypocreales	LC6928	/	/	Primer design	/
*Kylindria*	*peruamazonensis*	/	Reticulascaceae	Glomerellales	CBS 421.95	/	/	Primer design & qPCR test	-
*Leptosphaeria*	*spegazzinii*	/	Leptosphaeriaceae	Pleosporales	LC11735	/	/	Primer design & qPCR test	-
*Monilochaetes*	*guadalcanalensis*	/	Australiascaceae	Glomerellales	CBS 346.76	/	/	Primer design & qPCR test	-
*Musicillium*	*theobromae*	/	Plectosphaerellaceae	Glomerellales	CBS 968.72	/	/	Primer design & qPCR test	-
*Penicillium*	*chrysogenum*	/	Aspergillaceae	Eurotiales	LC11508	/	/	qPCR test	-
*Phoma*	*delphinii*	/	Didymellaceae	Pleosporales	CBS 134.96	/	/	Primer design & qPCR test	-
*Phoma*	*segeticola*	/	Didymellaceae	Pleosporales	LC1636	/	/	Primer design & qPCR test	-
*Phyllosticta*	*parthenocissi*	/	Phyllosticataceae	Botryosphaeriales	CBS 111645	/	/	Primer design & qPCR test	-
*Plectosphaerella*	*alismatis*	/	Plectosphaerellaceae	Glomerellales	CBS 113362	/	/	Primer design & qPCR test	-
*Plectosphaerella*	*populi*	/	Plectosphaerellaceae	Glomerellales	CBS 139623	/	/	Primer design & qPCR test	-
*Pseudocercospora*	*pini-densiflorae*	/	Mycosphaerellaceae	Mycosphaerellales	CBS 125139	/	/	Primer design	/
*Reticulascus*	*clavatus*	/	Reticulascaceae	Glomerellales	CBS 125296	/	/	Primer design & qPCR test	-
*Septoria*	*linicola*	/	Mycosphaerellaceae	Mycosphaerellales	CBS 502.50	/	/	Primer design & qPCR test	-
*Sodiomyces*	*alkalinus*	/	Plectosphaerellaceae	Glomerellales	CBS 110278	/	/	Primer design & qPCR test	-
*Sodiomyces*	*tronii*	/	Plectosphaerellaceae	Glomerellales	CBS 137618	/	/	Primer design & qPCR test	-
*Sporoschismopsis*	*angustata*	/	Reticulascaceae	Glomerellales	CBS 136360	/	/	Primer design & qPCR test	-
*Stachylidium*	*bicolor*	/	Reticulascaceae	Glomerellales	CBS 121802	/	/	Primer design & qPCR test	-
*Verticillium*	*dahliae*	/	Plectosphaerellaceae	Glomerellales	LC11567	/	/	Primer design & qPCR test	-

Notes: ^1^ The name of the species complex to which *Colletotrichum* species belong. ^2^ BCC: BIOTEC Culture Collection, National Center for Genetic Engineering and Biotechnology (BIOTEC), Khlong Luang, Pathumthani, Thailand; BRIP: Queensland Plant Pathology Herbarium, Australia; CBS: Culture collection of the Westerdijk Fungal Biodiversity Institute, Utrecht, The Netherlands; CGMCC: China General Microbiological Culture Collection Center, Institute of Microbiology, Chinese Academy of Sciences, Beijing, China; IMI: International Mycological Institute, CABI-Bioscience, Egham, Bakeham Lane, United Kingdom; LC: personal culture collection of Prof. Lei Cai, housed in the Institute of Microbiology, Chinese Academy of Sciences; MAFF: Ministry of Agriculture, Forestry and Fisheries, Tsukuba, Ibaraki, Japan; MFLU (CC): Mae Fah Luang University Culture Collection. ^3^ NCBI blast: Using the *Colletotrichum* 28S sequence corresponding to our designed specific primers and probes as the target, we performed a BLAST search in the NCBI genome database. The sequences with the 100% similarity were all identified as *Colletotrichum* spp. Primer design: Used for designing specific primer and probe. qPCR test: Used for testing the specificity of primer and probe. ^4^ +: Positive result in the qPCR test; -: negative result in the qPCR test; /: not tested.

### 2.3. Specificity Assessment of the TaqMan qPCR Assay

The specificity of the assay was empirically evaluated using genomic DNA from 78 fungal isolates (indicated as “Primer design & qPCR test” and “qPCR test” in the “Usage” column of Table 1). Sterile distilled water served as a blank control. Real-time PCR was performed using an ABI PRISM 7900HT Fast Real-Time PCR System (Applied Biosystems Company, Carlsbad, CA, USA). The 20 μL reaction mixture consisted of 10 μL of 2× TaqMan^®^ Universal PCR Master Mix, 0.5 μL each of forward primer CLF (10 μmol/L), reverse primer CLR (10 μmol/L), and probe CLP (10 μmol/L), 1 μL of template DNA, and 7.5 μL of sterile water. Thermal cycling conditions were: 95 °C for 10 min, followed by 40 cycles of 95 °C for 15 s and 60 °C for 60 s. Three technical replicates were performed for each sample.

### 2.4. Optimization of qPCR Conditions

Genomic DNA from *C. kahawae* (IMI 319418) was used as the template to optimize the qPCR assay. A blank control containing sterile distilled water was included in each run. First, primer concentrations were optimized by testing a gradient of forward and reverse primer concentrations (0.1, 0.2, 0.3, 0.4, 0.5, 0.6, 0.7, 0.8, 0.9, and 1.0 μmol/L) while keeping other component concentrations constant. The optimal primer concentration was selected based on the cycle threshold (Ct) values and the fluorescence intensity of the amplification curves. Subsequently, using the optimized primer concentrations, the probe concentration was optimized by testing a range from 0.1 to 1.0 μmol/L. Three technical replicates were performed for each condition. The thermal cycling conditions were identical to those described in the specificity assessment.

### 2.5. Sensitivity and Standard Curve Analysis

To assess sensitivity, genomic DNA from *C. siamense* (CGMCC 3.14193 = CBS 130420) was serially diluted 10-fold using RNase-free ddH_2_O, ranging from 68 ng/μL to 6.8 fg/μL. The optimized reaction system was used: 20 μL reaction volume containing 10 μL of 2× TaqMan^®^ Universal PCR Master Mix, 0.7 μL of forward primer CLF (10 μmol/L), 0.7 μL of reverse primer CLR (10 μmol/L), 0.5 μL of probe CLP (10 μmol/L), 1 μL of template DNA, and 7.1 μL of sterile water. The cycling conditions were: 95 °C for 10 min, followed by 40 cycles of 95 °C for 15 s and 60 °C for 60 s. Three replicates were performed for each dilution.

A standard curve was generated using the 10-fold dilution series of *C. siamense* DNA (dynamic range: 68 ng/μL to 68 fg/μL). The amplification efficiency (E) was calculated using the equation E = (10^(−1/slope)^ − 1) × 100. The slope and linearity (coefficient of determination, R^2^) were also determined.

### 2.6. Validation with Mock Environmental Samples

To evaluate the assay’s robustness and exclude potential interference from non-target species in complex matrices, two types of mock environmental samples were prepared using fungal genomic DNA. Mock Sample 1 (Non-target Cocktail): Prepared by pooling 1 μL of DNA from each of the 27 non-*Colletotrichum* strains (labeled as “qPCR test” or “Primer design & qPCR test” in Table 1) into a single tube. A 1 μL aliquot of this mixture was used as the template. Mock Sample 2 (Target Spiked in Non-target Cocktail): Prepared by transferring 10 μL of Mock Sample 1 to a new tube and adding DNA from 15 randomly selected *Colletotrichum* spp (1 μL each, as listed in Table 1). A 1 μL aliquot of this final mixture was used as the template.

Genomic DNA of *C. kahawae* (IMI 319418) served as a positive control, *Chordomyces antarcticus* (CBS 120045) as a negative control, and sterile distilled water as a blank control. The qPCR was performed using the optimized reaction conditions described above, with three technical replicates for each reaction.

## 3. Results

### 3.1. Specificity of the Colletotrichum spp.-Specific qPCR TaqMan Assay

**Figure 2:**
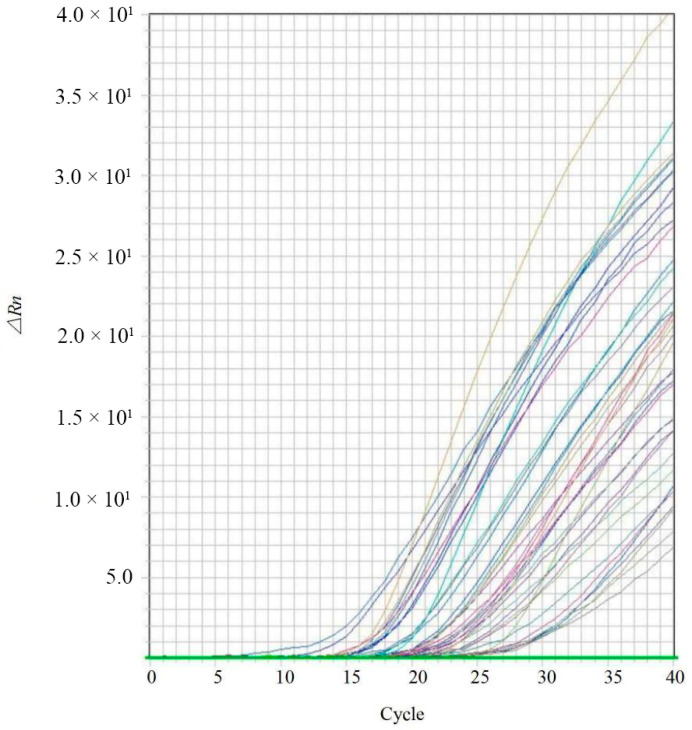
Real-time PCR amplification curves for the specific detection of *Colletotrichum* spp. The specificity was evaluated by testing the designed primers and probe against genomic DNA from target *Colletotrichum* isolates and various non-target fungal species. The amplification curves illustrate positive fluorescent signals exclusively for *Colletotrichum* spp., while no signals (flat lines) were observed for non-target species. The *X*-axis represents the cycle values, and the *Y*-axis represents the baseline-corrected normalized fluorescence (ΔRn).

Specificity was evaluated using the 78 fungal isolates listed in Table 1. The results demonstrated that positive amplification signals were observed exclusively in *Colletotrichum* samples (Figure 2), while no amplification was detected in any non-target species. Variations in the observed cycle threshold (Ct) values were attributed to the lack of DNA concentration normalization across samples during specificity testing. Furthermore, one of the 69 bp target amplicon sequences of *Colletotrichum* spp. was queried against the NCBI Genome database using BLASTn. The top hits were consistently identified as *Colletotrichum* species. Subsequent local alignment of at least 200 retrieved sequences from NCBI revealed 100% identity with the target amplicon, confirming the high intrageneric conservation of the designed markers.

### 3.2. Optimization of qPCR Conditions

**Figure 3:**
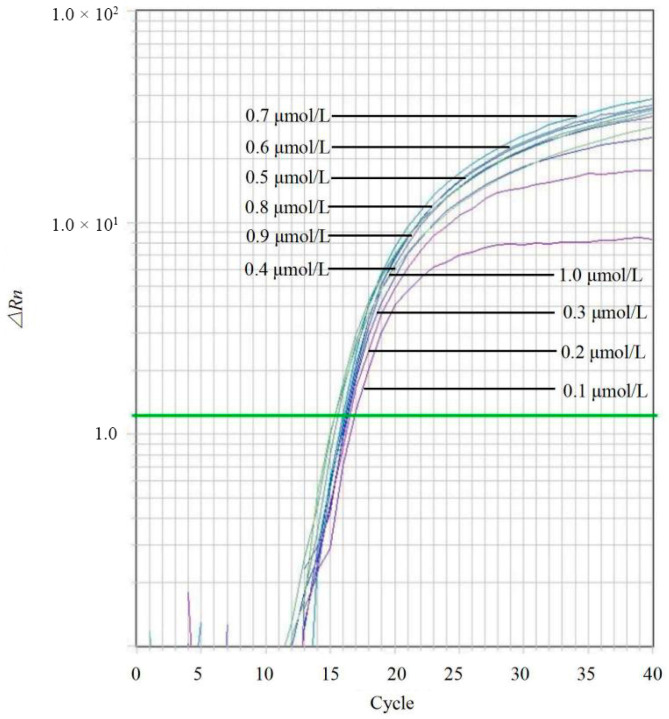
Optimization of primer concentrations for the *Colletotrichum*-specific TaqMan qPCR assay. A range of primer concentrations (0.1, 0.2, 0.3, 0.4, 0.5, 0.6, 0.7, 0.8, 0.9, and 1.0 μmol/L) was evaluated while keeping the probe concentration and template DNA constant. Each curve represents the mean amplification plot of a specific primer concentration. The green line represents the threshold line automatically generated by the real-time PCR instrument software.

**Figure 4:**
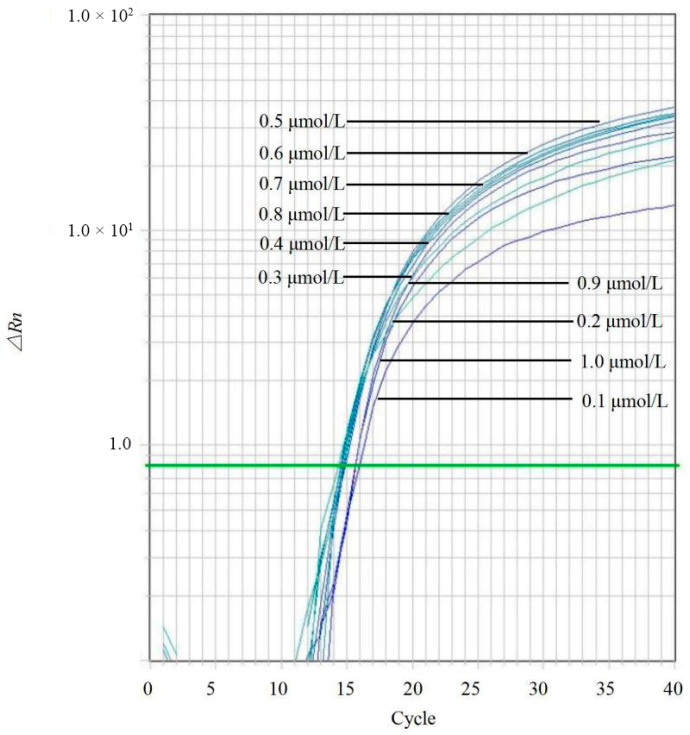
Optimization of probe concentrations for the *Colletotrichum*-specific TaqMan qPCR assay. The assay was performed using varying probe concentrations (0.1, 0.2, 0.3, 0.4, 0.5, 0.6, 0.7, 0.8, 0.9, and 1.0 μmol/L) to identify the concentration yielding the highest fluorescence signal and lowest Ct value. Curves correspond to the respective probe concentrations as indicated in the legend. The green line represents the threshold line automatically generated by the real-time PCR instrument software.

Positive amplification signals were observed at all primer concentrations ranging from 0.1 to 1.0 μmol/L (Figure 3). However, the reaction containing 0.7 μmol/L of primers yielded the highest fluorescence signal (∆Rn) and the lowest Ct values, indicating optimal amplification efficiency. Based on the optimized primer concentration, the probe concentration was subsequently tested. The results indicated that a probe concentration of 0.5 μmol/L produced the maximum ∆Rn and minimum Ct values (Figure 4). Consequently, the optimized qPCR reaction system (20 μL total volume) was established as follows: 10 μL of 2× TaqMan^®^ Universal PCR Master Mix, 0.7 μL of forward primer CLF (10 μmol/L), 0.7 μL of reverse primer CLR (10 μmol/L), 0.5 μL of probe CLP (10 μmol/L), 1 μL of template DNA, and 7.1 μL of sterile water.

### 3.3. Sensitivity of Real-Time Fluorescent PCR for Detection of Colletotrichum spp.

**Figure 5:**
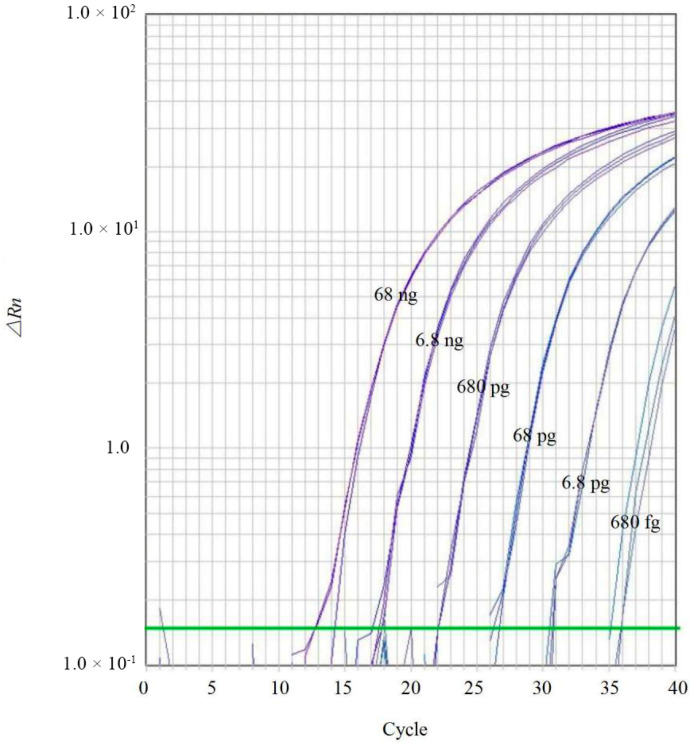
Sensitivity of the TaqMan qPCR assay using 10-fold serial dilutions of *C. siamense* genomic DNA as templates. The amplification curves correspond to DNA concentrations ranging from 68 ng/μL to 680 fg/μL per reaction. Each concentration was tested in triplicate to ensure reproducibility. The green line represents the threshold line automatically generated by the real-time PCR instrument software.

The sensitivity of the assay was evaluated using a ten-fold serial dilution of genomic DNA from the ex-type strain of *C. siamense* (CGMCC 3.14193), ranging from 68 ng/μL to 6.8 fg/μL. Positive amplification curves were consistently obtained at concentrations ≥680 fg/μL, establishing the limit of detection (LOD) at 680 fg/μL (Figure 5).

**Figure 6:**
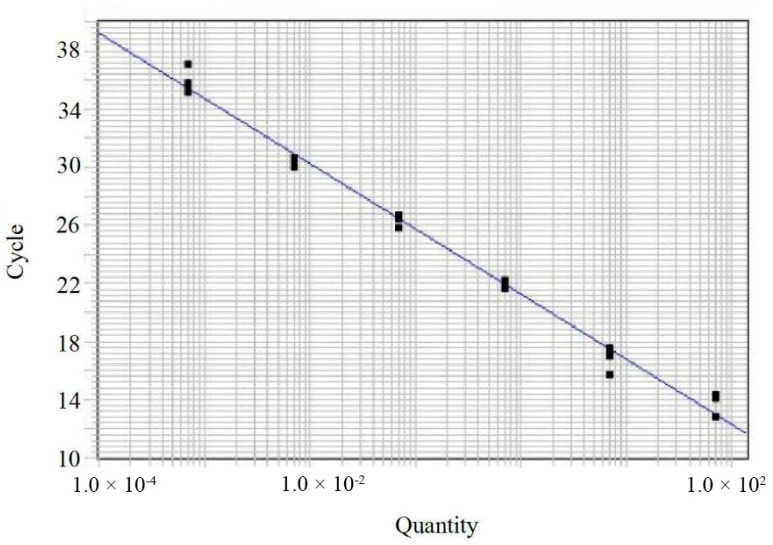
Standard curve of the TaqMan qPCR assay for the quantification of *Colletotrichum* DNA. The standard curve was generated by plotting the Ct values (cycle, *y*-axis) against the logarithm of the genomic DNA concentrations (quantity, *x*-axis) from *C. siamense*. Each data point represents the mean Ct value derived from three independent replicates.

There was a strong inverse linear relationship between the Ct values and the logarithm of the template DNA concentrations. The standard curve generated from the triplicates showed a linear regression equation of *y* = −4.49x + 21.29, with a coefficient of determination (*R^2^* = 0.9922) (Figure 6). These data demonstrate that the established assay possesses high reproducibility and reliable amplification performance within the tested dynamic range.

### 3.4. Detection of Colletotrichum spp. in Mock Environmental Samples

The specificity and practical applicability of the developed TaqMan qPCR assay were further validated using mock environmental samples. Positive amplification curves were detected in the positive control and Mock Sample 2 (containing *Colletotrichum* DNA). In contrast, no fluorescence signals were observed in the negative control, the blank control, or Mock Sample 1 (containing only non-*Colletotrichum* fungal DNA). These results confirm the assay’s ability to accurately detect *Colletotrichum* species within complex fungal communities without cross-reactivity.

## 4. Discussion

Specificity is the prerequisite for any reliable molecular diagnostic assay [24]. In this study, the specificity of the designed primer-probe set was rigorously challenged against a comprehensive panel representing the fungal diversity typically encountered in agricultural and clinical ecosystems. By including representatives from 14 *Colletotrichum* species complexes [19] and, crucially, species from the phylogenetically sister families—Australiascaceae, Plectosphaerellaceae, and Reticulascaceae [25]—we effectively excluded cross-reactivity with proximate taxa. The absence of false-positive signals in these closely related groups, as well as in other common phytopathogens (e.g., members of Botryosphaeriales, Chaetosphaeriales, Diaporthales, Eurotiales, Hypocreales, Mycosphaerellales, and Pleosporales), confirms that the selected 28S rDNA region offers sufficient phylogenetic resolution to discriminate the genus *Colletotrichum* from other fungal lineages.

In the initial phase of assay development, the ITS region was evaluated as a potential target. However, achieving a balance between intergeneric specificity and intrageneric conservation proved challenging due to the high variability of ITS sequences within the genus and the limitations of ITS as a sole marker for certain *Colletotrichum* lineages [26]. Consequently, we shifted our focus to the 28S rDNA region, which generally exhibits superior stability at the genus level [27]. A significant hurdle was the paucity of *Colletotrichum* 28S sequences in public repositories, as this region is not routinely employed in the taxonomy of this genus compared to multi-locus sequence analysis genes like *gapdh* or *tub2* [8]. To bridge this gap, we generated 28S sequences for multiple *Colletotrichum* strains via in-house sequencing. We then implemented a robust dual-validation strategy, combining empirical qPCR testing with in silico verification. This comprehensive approach confirmed the assay’s efficacy for 14 of the 17 species complexes described by Liu et al. [19].

The remaining three species complexes (*C. agaves*, *C. graminicola*, and *C. bambusicola*) could not be experimentally validated due to the unavailability of reference strains at the time of the study. To evaluate the assay’s potential performance for these groups, sequence alignment analysis was conducted, which identified a single nucleotide polymorphism (SNP), specifically a T-to-C transition, in the central region of the reverse primer binding site. Within the framework of TaqMan chemistry, a single central mismatch generally permits target amplification, although it may delay the threshold cycle by 0.5–2 cycles. While this may slightly influence quantification accuracy, it does not typically compromise qualitative detection [28]. This theoretical resilience was empirically supported in the present study; several species used for validation, such as *C. aracearum* (CGMCC 3.14982), *C. boninense* (CGMCC 3.14356), and *C. falcatum* (CBS 147945), harbor similar mismatches in their reverse primer binding regions (Figure 1). Despite these sequence variations, all such taxa were accurately detected, demonstrating that the assay maintains high diagnostic sensitivity and is capable of providing universal coverage for the genus *Colletotrichum*.

Previous molecular detection efforts for *Colletotrichum* have predominantly focused on species-specific assays [20,21,29,30]. While valuable for precise taxonomic identification, these narrow-spectrum assays are often constrained by the genus’s frequent taxonomic reclassifications and the rapid emergence of cryptic pathogenic species [5]. A primer set designed for a specific species today may become taxonomically ambiguous due to future revisions. In contrast, the genus-specific assay established here targets the conserved 28S rDNA region, functioning as a stable and robust “universal” screening tool. This capability is particularly advantageous in phytosanitary inspection, where the primary objective is to rapidly confirm the presence of *Colletotrichum* infection to trigger immediate control measures, rather than to determine the specific cryptic species immediately.

The high sensitivity and broad specificity of this assay have significant implications for both agriculture and public health. In phytosanitary contexts, *Colletotrichum* species are notorious for establishing latent infections in asymptomatic plant tissues, rendering visual inspection at customs borders ineffective [31]. With a limit of detection as low as 680 fg, our TaqMan qPCR assay enables the interception of low-titer pathogen DNA in asymptomatic germplasm, serving as a powerful early warning tool.

Furthermore, the significance of this method extends to clinical diagnostics. As *Colletotrichum* species are increasingly recognized as opportunistic human pathogens causing keratitis and subcutaneous infections [9,14,17], rapid diagnosis is vital. Since first-line antifungal treatments often differ between fungal genera but are consistent within the genus, this assay allows for the rapid confirmation of *Colletotrichum* etiology, thereby facilitating timely clinical decision-making and the initiation of appropriate antifungal therapy [32].

Ensuring robust performance in complex biological matrices is essential for the practical utility of any diagnostic assay [33]. Although this study utilized simulated samples, they were specifically engineered to mimic the complexity of environmental material by incorporating a diverse background of non-target genomic DNA. This mixture comprised several common phytopathogenic genera, including *Cercospora*, *Curvularia*, *Diaporthe*, *Leptosphaeria*, *Plectosphaerella*, *Phoma*, and *Verticillium*, as well as the trans-kingdom pathogen *Fusarium* [34,35]. The lack of cross-reactivity within these complex backgrounds, alongside in silico BLASTn analysis confirming no significant homology between the target sequence and host genomes, highlights the high analytical specificity of the assay. Furthermore, the ability of this qPCR method to directly identify *Colletotrichum* DNA from environmental samples without the requirement for prior fungal isolation offers a significant advantage for rapid diagnostics. This bypassing of traditional culture-based methods can reduce the diagnostic timeline from several days to about one hour, providing a reliable tool for both agricultural and clinical detection of *Colletotrichum* infections. Moreover, although the calculated amplification efficiency of this assay was 66.7% (slope = −4.49), falling outside the ideal range for precision absolute quantification, the high correlation coefficient (*R^2^* = 0.9922) ensures sufficient reliability for qualitative screening and semi-quantitative analysis in diagnostic applications.

## Data Availability

The GenBank accession numbers for the public data used in this study are listed in Table 1, further inquiries can be directed to the corresponding author.
